# Evolutionary perspective of drug eluting stents: from thick polymer to polymer free approach

**DOI:** 10.1186/s13019-022-01812-y

**Published:** 2022-04-04

**Authors:** Sadia Hassan, Murtaza Najabat Ali, Bakhtawar Ghafoor

**Affiliations:** Department of Biomedical Engineering and Sciences (BMES), School of Mechanical and Manufacturing Engineering (SMME), National University of Sciences and Technology (NUST), Islamabad, Pakistan

**Keywords:** Bare metal stents, Angioplasty, Xience, Sirolimus, Stents, Cardiovascular disease

## Abstract

**Background:**

Introduction of Bare Metal Stents (BMS) was itself a revolutionary step in the history of the medical industry; however, Drug Eluting Stents (DES) maintained its superiority over BMS in every aspect from restenosis rate to late lumen loss. The reason behind the magnanimous position of the DES in the stent market is the degree of improvement with which it evolves. New and better stents come into the market every year, surpassing their predecessors by many folds.

**Literature review:**

This review paper discusses the journey of DES with supporting clinical trials in detail. In the first generation, there were stainless-steel stents with thicker coatings. Although they had superior results compared to BMS, there was still room for improvement. Afterward came the second-generation stents, which had superior metal platforms with thinner struts and thin coatings. The drugs were also changed from Paclitaxel and Sirolimus to Zotrolimus and Everolimus. These stents performed best; however, there was an issue of permanent coating, which remained intact over the stent surface after complete drug elution and started to cause issues in longer-term studies. Hence, an improved version of DES was introduced to these permanent coatings called the third generation of drug eluting stents, which initially utilized biodegradable polymer and ultimately moved towards polymer free drug coatings. This generation has introduced a unique amalgam of technologies to achieve its polymer free coatings; however, researchers have numerous prospects of growth in this field. This review paper highlights the major coups of stent technology evolution from BMS to DES, from thick polymeric coatings to thin coatings and from durable polymers to polymer free DES.

**Conclusion:**

In conclusion, though the medical industry promptly accepted BMS as the best treatment option for cardiovascular diseases; however, DES has provided even better results than BMS. In DES, the first and second generation has ruled the technology for many years and are still on the shelves. Still, the issues aroused due to durable polymer shifted the attention towards biodegradable drug eluting stents, the third generation growing rapidly. But the scientific community has not restricted themselves and is investigating bioresorbable stents that completely eliminate the polymer intervention in drug eluting stent technology.

## Introduction

The lifestyle of the twenty-first century has reduced the level of physical activity of a common man and has contributed to the development of many lethal diseases, which were rare a few decades ago. These diseases include diabetes, cancer, obesity, and the most common one is ischemic heart disease. Cardiovascular diseases (CVDs) are caused due to accumulation of fatty acids, diabetes, high blood pressure, smoking, and physical inactivity leading to plaque formation. Percutaneous coronary intervention (PCI) and stent technology have been come into play to curb this global issue. In PCI, a stent is crimped over the deflated balloon and deployed at the target site using the catheter's delivery device. The deflated balloon is inflated once the target site expands the crimped balloon squeezes the plaque, opening the obstruction to the flow of blood to maintain its flow. Thus, metallic stent placement is the most revolutionary medical intervention and rapidly accepted treatment option for CVDs. Since the advent of stent technology, researchers have been in a constant struggle to improve PCI outcomes. Though stent technology was a breakthrough in the field of surgery, there are some drawbacks are associated with it i.e., in-stent restenosis and increased risk of thrombosis aroused due to injury caused to the endothelium during the procedure. The neointimal hyperplasia and repeated revascularization due to BMS implantation lead to the introduction of a pharmaceutically active agent that is applied onto the BMS in coatings with a polymer as drug carrier called DES to lower the risks posed by BMS. Although DES was carefully designed to reduce stent thrombosis, the risk of late in-stent thrombosis and restenosis is seen in DES clinical trials. In this regard, continuous research is on-going to improve the DES technology by altering its platform, polymers used, and drug incorporated, which gave the medical world various improvised versions of DES technology to meet the demands of improved quality of life and reduce the rate of deaths due to myocardial infarction. This review aims to discuss the various generations of DES and how they are evolved to encompass the issues aroused by previous BMS and DES.

## Bare metal stents

In 1963, a group of surgeons performed an abdominal aortogram of an occluded iliac artery, and they accidentally recanalized it during the procedure. This accidental discovery led them to perform the first percutaneous transluminal peripheral angioplasty in 1964 with the help of a catheter. Scientists and cardiologists kept trying, and in 1977, the first balloon percutaneous transluminal coronary angioplasty was successfully performed. The success of these experiments opened the gate for discoveries, which led to the development of the first coronary stent (self-expandable) in 1986. In 1987, the first balloon expandable stent was developed, which became the first product to get the approval of the FDA in the coronary stent market [1]. The metallic mesh with a specific geometric structure acts as a scaffold in vascular structure and opens up the narrowed areas maintaining the blood flow at the specified area. Thus, the stent implantation is the permanent treatment solution in which the stent stays in the target area. For the first time, the stent was developed by Palmaz and Schatz [Johnson & Johnson, USA] to achieve superior results than POBA (Plain Old Balloon Angioplasty) by giving a permanent and long term solution as compared to POBA, which is a temporary treatment option and its results as a short term only. In this regard, In 1994, two clinical trials, the BENESTENT, and the STRESS were performed. The results proved the superiority of BMS over POBA because in POBA balloon is inserted into the body to compress the plaque, which opens up the lumen for blood flow. Still, lesion regrows or expands after some time, putting the body in the same old situation resulting in a second intervention.

On the other hand, the stent stays at the target sites and keeps the artery open providing a better and long lasted solution. The BENESTENT trial was a multicentric trial that took place at 28 different medical centers, including 520 patients, and among them, 259 received stents [2]. The clinical outcomes demonstrated no significant difference in mortality rate, myocardial infarction, and stroke; however, the rate of revascularization decreased by 10% in the stent group compared to the balloon group [3]. The results of the STRESS trial (407 patients at 20 different medical centres) were published in 1995, which demonstrated results similar to the BENESTENT trial with no significant difference in major cardiac events between the patients who received stent who underwent balloon angioplasty [4]. The one year follow up of the STRESS I trial demonstrated no occurrence of coronary events; however, the incidence of target lesion revascularization (TLR) was 10% in the stent group compared to 15% in the balloon angioplasty group [5]. The STRESS II trial was conducted on 598 patients. One year follow–up demonstrated that in the stent group, 78% of patients had no incidence of any major cardiac event. One year follow-up demonstrated that in the stent group, 78% of patients had no incidence of any major cardiac event compared to 67% of the balloon angioplasty group. The the target lesion or vessel revascularization rate was 16% in the stent group vs 26% in the balloon angioplasty group [6]. STRESS III study was conducted to observe the effects of increased pressure in balloon angioplasty compared to stenting. Results of one year follow up demonstrated that increased pressure does not have effect on the clinical outcomes.

Though at the early stages of BMS, BMS was performing better than any other minimally invasive strategy. Still, in the very long term, through clinical studies, it was revealed that BMS is associated with high rates of restenosis, which are unacceptable for CAD treatments. During stent implantation, vessel injury is caused while clearing the plaque, which triggers the inflammatory response promoting fibroblast growth and smooth muscle cell proliferation resulting in neointimal hyperplasia. As BMS is only the metallic mesh structure without any additive active pharmaceutical agent to cater to the root cause of restenosis, the plaque,, after some time, the stent becomes occluded by the ingrowth of plaque and neointimal hyperplasia. Moreover, Bare metal stents do not contain any anti-inflammatory agents, which can reduce the inflammatory response resulting in higher restenosis rates [7]. BMS is currently used to treat larger vessels where restenosis rates are lower [8]. BMS is still used in 20% of coronary angioplasty procedures [9]. I. M. Singh et al. conducted a study on all the angioplasty procedures done between 1999 and 2007 at Cleveland Clinic. Total 706 procedures were shortlisted in which 362 patients were implanted with DES and 344 patients with BMS. The rate of death and TLR was 8% and 3% in the DES group vs 24% and 8% in the BMS group [10]. Another study was conducted to demonstrate the effects of DES on neointimal hyperplasia compared to BMS. The results demonstrated that the hyperplasia volume was 5.4 mm3 in DES compared to 35.9 mm^3^ in BMS [11]. Several other clinical trials have proven reduced rates of restenosis and neointimal hyperplasia in DES compared to BMS [12].

## Drug eluting stents (DES)

To solve the problems of BMS, scientists evolved the stent platform (including thinner stent struts and new metallic platforms) and stent treatment principles (drug coatings). These drug coated stents are termed drug eluting stents (DES). In addition to a metallic platform like BMS, DES has two more components: Pharmaceutical agent and carrier of a pharmaceutical agent. An ideal pharmaceutical agent for CAD should be an antiproliferative and/or immunosuppressant. It should attack the proliferative response without any systematic side effects. An ideal pharmaceutical agent carrier should be able to store the drug effectively and control its release according to the desired rate and provide release kinetics that are effective to inhibit restenosis. Lastly, an ideal stent platform should be flexible, thin, and appropriate for coatings.

Scientists tried to incorporate all these attributes in their first-generation stents and got exciting results. Since then, there has been a continuous battle to improve the stent designs, thickness, platform, drugs, and release kinetics. So far, researchers have been successful in developing stents with thinner struts, a different metallic platform consisting of various alloys, and numerous drugs, polymers, and their combinations. These attributes were introduced gradually, and each generation of DES emerged with new and improved features dominating the others. DES has become the mainstream therapy of coronary artery stenosis due to the less expected rate of in-stent restenosis outdating the brachytherapy. In the later sections of this paper, various DES and their profound features are discussed with clinical trials outcomes as a supporting argument for the new entry of DES.

## First generation DES

Studies have demonstrated that about 20% of patients treated with BMS had high rates of restenosis and target lesion revascularization (TLR), resulting in re-stenting within 12 months of implantation [13]. The complications of BMS led to the use of anti-inflammatory, anti-proliferative, and anti-restenosis drugs on the stents. The drug release kinetics were controlled with the help of polymers and adhesives. These drugs and polymers evolved with time, and currently, four different generations of stents are available over the shelf. The principal features of each generation are listed in Table 1.

**Table 1:** Profound features of three generations of drug eluting stent

Generations		Platform	Strut thickness	Polymer	Polymer type	Drugs	Commercially available DES
1st		Stainless Steel	> 130	SIBS PEVA PBMA	Permanent	Sirolimus Paclitaxel	Cypher Taxus
2nd		CoCr PtCr	80 µm to 100 µm	PVDF-HFP BioLinks	Permanent	Everolimus Zotrolimus	Xience Endeavour Resolute
3rd	A	CoCr PtCr	55 µm to 85 µm	PLA PGLA	Biodegradable	Sirolimus Everolimus Biolimus	YukonPC Biomatrix Orsiro Ultimaster Synergy
	B	CoCr Stainless steel	70 µm to 90 µm	Polymer free	–	Sirolimus Probucol	YukonChrome ISAR
4th		PLLA Magnesium	130–150	PLLA PDLLA	Biodegradable	Sirolimus Novolimus Everolimus	Absorb Absorb-GT1 MeRes Magmaris

**Fig. 1:**
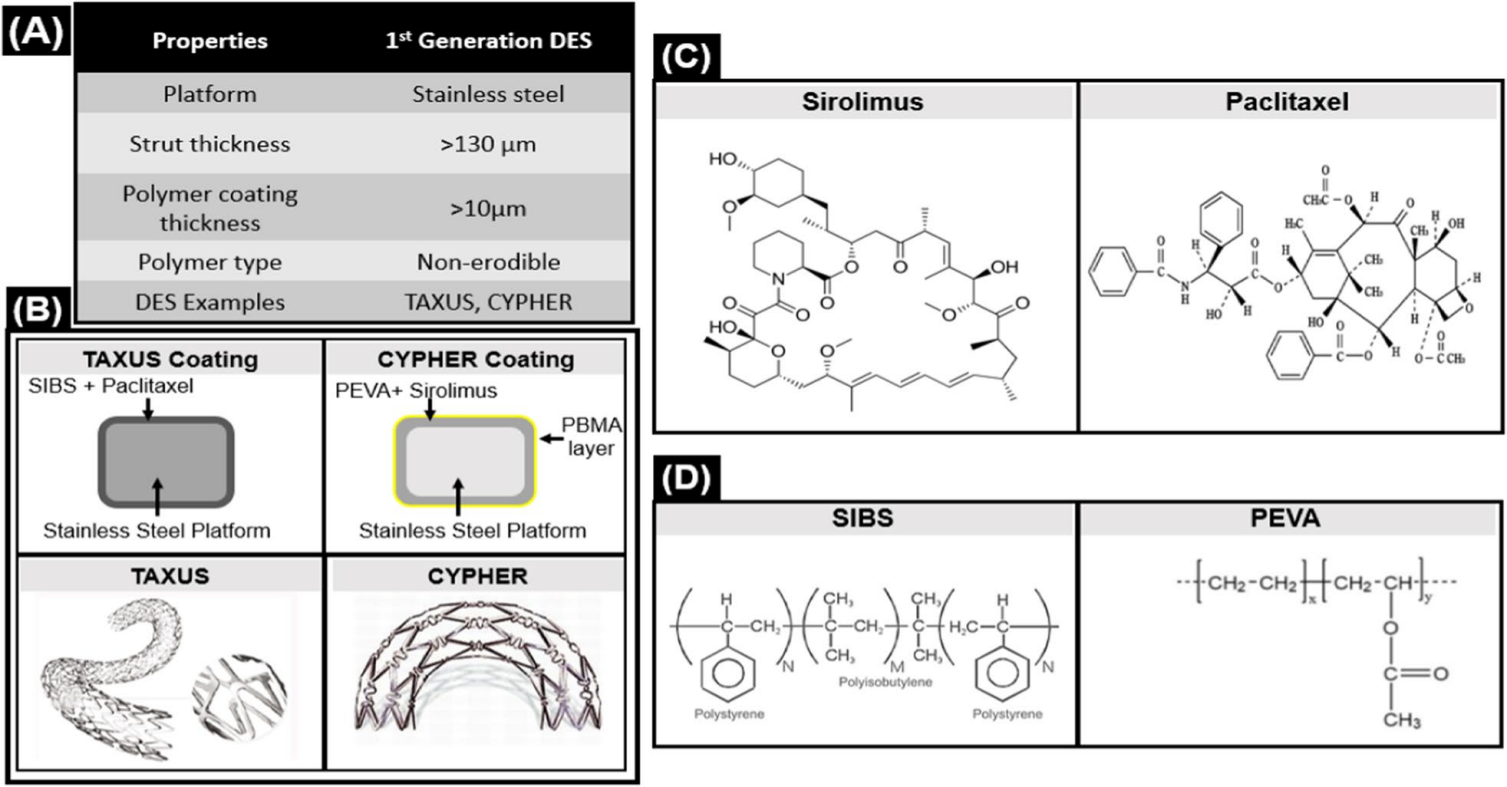
Attributes of first-generation DES **A** Overall properties of 1st generation DES **B** Coating and design of DES from 1st generation **C** Drugs used in 1st generation DES **D** Polymers used in 1st generation DES

The first generation of drug eluting stents was introduced in 2002 when Cordis/Johnson & Johnson received a CE mark for Cypher Sirolimus Eluting Stent. Next year, Boston Scientific received a CE mark for another DES named Taxus Express™ and launched the product in 2004. These two stents make the first generation of stents and are considered pillars of DES technology. The attributes of this generation are summed up in Fig. 1:

### Cypher

The first ever drug eluting stent floated in the medical market was Cypher with Stainless Steel platform (strut thickness = 132 μm), Sirolimus eluting stent (shown in Fig. 1). Cypher is coated with a unique PEVA and Sirolimus combination along with a PBMA base coat. The total coating thickness is 12.6 μm. The layers of coating are explained below:

1. The first layer is a mixture of sirolimus (67:33%), PEVA (polyethylene-co-vinyl acetate), and PBMA (poly n-butyl methacrylate)
2. The second layer is composed of drug free PBMA [14]
3. The third layer is Parylene C

Sirolimus is a lipophilic antibacterial and anti-fungal drug. It has properties of anti-proliferation and immunosuppressants which made it a suitable candidate for the stent coating [15]. The 80% sirolimus is released within 30 days after stent implantation, while complete release occurs in 90 days.

The first non-randomized clinical evaluation of CYPHER DES was conducted on 30 patients for two years. This trial demonstrated 6.7% MACE, 0% TLR, and thrombosis. After the success of the first-in-man evaluation, two prospective, double-blind, multi-centric, and randomized trials SIRUS and RAVEL, were conducted. One-year results of the SIRUS trial (n = 1058) demonstrated 2.4% in hospital MACE and 6% out of hospital MACE. During the trial, zero incidences of thrombosis were reported with a single incidence of TLR. The RAVEL trial (n = 238) demonstrated 2.5% in hospital and 7.5% out of hospital MACE for two years studies. Throughout the study, only single incidence of thrombosis and three incidences of TLR were observed. These trials showed superior performance of sirolimus eluting first compared to other drug eluting stents for two years of follow up.

### Taxus express™

After Cypher, Taxus was introduced with a different pharmaceutical agent into the market. Taxus Stent is a paclitaxel eluting stent of stainless steel with 140 um thick sturts, as shown in Fig. 1. It has a 19.6 μm thick single layer coating of a mixture of Paclitaxel and SIBS (poly(styrene-block-isobutylene-block-styrene). Paclitaxel is a naturally derived anti-proliferative drug coated on the Taxus stent. It is an antineoplastic drug widely used in the treatment of cancer, and the FDA approved it in 1999 for the treatment of breast cancer [16].

SIBS is a non-degradable polymer that allows a sustained and slow drug release from the matrix. SIBS polymer can last up to 2.5 years without degrading [17]. The release kinetics of Taxus suggested only 10% release of Paclitaxel within ten days and the remaining 90% of the drug remains part of polymer forever and released slowly [14].

TAXUS stent has been clinically studied in various trials. TAXUS I was the first randomized, multicentric trial of TAXUS DES, studied on 61 patients. One-year follow up demonstrated 3% MACE and zero incidences of thrombosis and TLR. This was a feasibility check study to learn about the effects of the amount of drug and release kinetics of drug formulation. The promising results led the stent to be studied on a larger population with complex lesions [18].

TAXUS II was a double-blind, randomized trial that was conducted in 15 countries at 38 different medical centres, including 536 patients. Two TAXUS DES (Slow Release TAXUS and Medium Release TAXUS were compared with BMS. The 5 years follow up proved the safety and superiority of TAXUS over BMS by demonstrating 22.5%, 16.6%, and 9.0% TVR, 18.4%, 10.3%, and 4.5% TLR and 7.6%, 20.4%, and 15.1% MACE in BMS, Slow Release TAXUS and Medium Release TAXUS respectively [19]. TAXUS stent has been widely studied clinically and has proven its safety and efficacy in TAXUS III and TAXUS IV.

TAXUS V clinical trial was a double blind and randomized trial which started in 2003 at 66 different medical centres in the USA. The nine months follow up demonstrated 12.1% TVR and 8.6% TLR in the Paclitaxel DES group as compared to 17% and 15.7% respectively in the BMS group. TAXUS stent proved its superiority on BMS until five years follow up [20].

### Cypher vs. Taxus

Stents of the first generation significantly reduced the restenosis rates and gave better results than the BMS. The effectiveness of both drugs has been proven clinically; however, a few studies suggested that sirolimus eluting stents (SES) are more effective than paclitaxel eluting stents (PES). In REALITY, clinical trial SES and PES were compared, and results exhibited that the rate of major cardiac adverse effect (MACE) was the same in both stents; however, In-stent late loss (ISR) was significantly lower in the sirolimus group (0.09 mm) than the paclitaxel group (0.31 mm). Also, the rate of stent thrombosis (ST) was higher with the PES (1.9%) as compared to SES (0.7%) [21].

The reason for better performance of SES can be linked to its coating system. According to scientific studies, drug release from drug eluting stents should be swift enough to plummet immediate restenosis and thrombus formation. After some time, the release should slow down to maintain the lesion healing. The drug release kinetics of Cypher is closest to the requirements, but Taxus has slow-release kinetics as it takes years to release 90% of drug from the matrix.

Despite providing better results than BMS, there were some issues such as chronic inflammation and delayed arterial healing associated with the first-generation stents, which led to the development of second-generation stents.

## Second generation stents

**Fig. 2:**
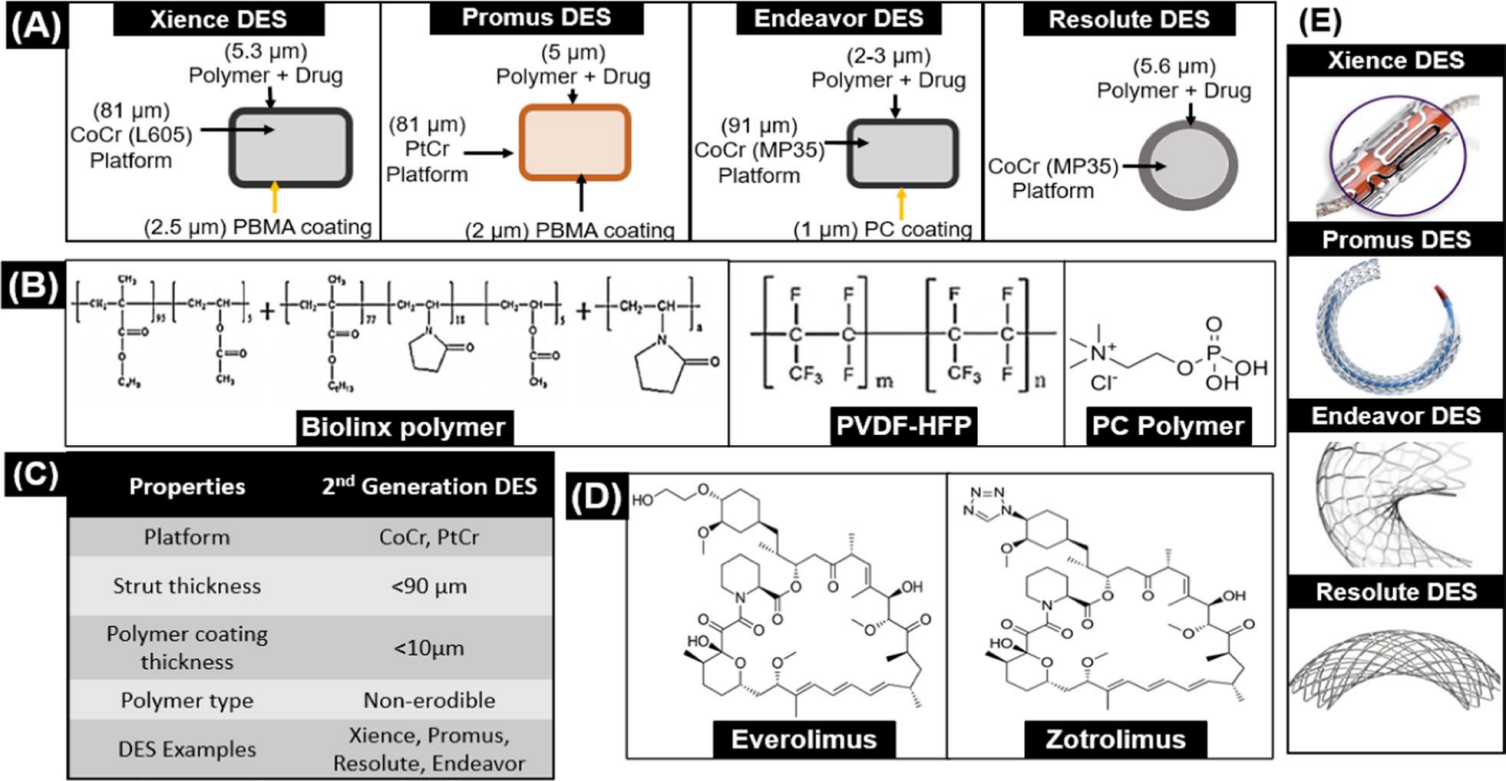
Attributes of 2nd generation DES **A** Commercially available 2nd generation DES **B** Polymers used in 2nd generation DES **C** Properties of 2nd generation DES **D** Drugs used in 2nd generation DES **E** Design of 2nd generation DES

The issues of the first generation steered the wheel of research groups towards better and improved products, which resulted in the introduction of stents having thinner struts, better polymeric coatings, and new drugs. The core characteristics [22] of second-generation DES are given in Fig. 2.

### Metallic platforms

The need for thinner struts was demonstrated in ISAR-STEREO Trial, conducted in 2001, where the stenosis rates of stents with same characteristics were compared; the only variable was strut thickness. ACS RX Multi-Link with strut thickness 50 µm and 100 µm were implanted in 651 patients. Outcome of this trial indicated that the mortality rate in the thin strut group was 1.5% as compared to 2.5% of the thick strut group. The results are given in the following Table 2.

**Table 2:** ISAR-STEREO trial outcome

Group	Strut thickness ( µm)	Mortality rate (%)	MI (%)	Restenosis (%)	Reference
Thin Strut (n = 325)	50	1.5	0.9	15	[23]
Thick Strut (n = 325)	140	2.5	1.2	25	

Thinner strut demonstrates better clinical results since these potentially reduce inflammation and vascular injury, accelerate endothelialization, and decrease neointimal proliferation and thrombogenicity by providing less contact surface for the body cells to elicit these responses [24]. The results of the ISAR trial started the debate of using stents with thinner struts because of better results obtained in the trial compared to stents with thicker struts. Moreover, it was the preference of the manufacturers. To further solidify the results, ISAR-STEREO-2 trial data (as given in Table 3) was published in 2003, which complimented the results of ISAR-STEREO.

**Table 3:** ISAR-STEREO trial outcome (6-month follow-up)

Group	Target vessel revascularization	Restenosis (%)	MI and death	Reference
Thin Strut (n = 309)	12.3	17.9	No significant difference in 1-year follow-up	[25]
Thick Strut (n = 302)	20.9S	31.5		

Both trials gave evidence-based knowledge that the thinner strut improves the stent quality and reduces the issues associated with it. The new metallic platforms for stent, Cobalt Chromium (CoCr) and Platinum Chromium (PtCr), were introduced for thinner struts to keep the other properties of the stent intact such as radial strength, recoil, etc. CoCr is a denser metal compared to Stainless steel with enhanced properties that are best suited for developing stent, as shown in Table 4. On the other hand, PtCr is a specialized base metal developed to improve stent quality. With CoCr and PtCr, strut thickness reduces up to 70 µm as compared to 130 µm of strut thickness in the case of stainless steel. Properties of Stainless steel, CoCr (mp35), CoCr (L605), and PtCr are given in Table 4.

**Table 4:** Properties of stent metals

Properties	Stainless steel 316L	CoCr (mp35)	CoCr (L605)	PtCr	Reference
Elastic Modulus (GPa)	190	233	243	203	[26]
Yield strength (MPa)	275	414	500	480	
Tensile strength (MPa)	535	930	1000	834	
Density (g/cm^3^)	7.9	8.4	9.1	9.9	

### Drugs

New drugs from the Limus family were introduced in the second-generation stents. The new drugs Zotarolimus and Everolimus are derivatives of Sirolimus. Both derivatives have the same structure as Sirolimus; however, they differ in the type of functional group.

Zotarolimus is a semisynthetic derivative of Sirolimus. Its mechanism of action is similar to Sirolimus, but chemical composition is different by the insertion of tetrazole ring at position 42 (substitution of the hydrophilic hydroxyl group) of the native structure [27]. This modification increases the lipophilic character of the drug and makes Zotarolimus most lipophilic and water-repellent among Sirolimus analogs. This modification gives Zotarolimus sustained and slow release kinetics instead of burst release [28].

Everolimus is the second derivative of sirolimus, and to date, Everolimus eluting stents (EES) are considered best among second generation stents. Like Zotarolimus, Everolimus is also a semi- synthesized hydroxyethyl ether derivative of Sirolimus whose modification has been done by the insertion of a 2-hydroxyethyl group at position 40 replacing hydroxyl group on the native structure. This modification makes Everolimus polar and lipophilic [29]. The benefits of Everolimus include better bioavailability, less clearance time, higher cellular residence time, and good absorption as compared to Sirolimus [30].

### Polymeric coatings

For DES, the nature of polymer and composition became the key factor since the release kinetics of the drug depends on it. Though the outcomes of the first generation of drug eluting stents were very encouraging, some issues like uncontrolled neointimal proliferation and inflammation were seen in first generation stents, and one of the main causes for eliciting these responses was the nature of the polymer. For better results, new and different types of polymers were introduced in the second generation of stents which are explained below:

- Abbott Laboratories introduced a polymer comprising of fluorinated copolymer poly(vinylidene fluoride)‐co‐hexafluoropropylene (PVDF-HFP). This fluorinated copolymer had the specialty to endure expansion by maintaining its elastic elongation [31].
- Medtronic introduced Phosphoryocholine polymer, composed of four different monomers of Methacrylate (2-methacryloyloxyethyl, lauryl methacrylate, trimethoxysilylpropyl methacrylate and 2-hydroxypropyl-methacrylate. This polymer is naturally present in our membrane. It is highly hydrophilic and does not activate inflammatory response [32].

### Commercially available second-generation stents

The second Generation of DES houses a large number of commercially available stents. The first Second Generation DES was Medtronic’s Endeavour which received FDA approval in 2004. Abbott’s Xience and Boston Scientific’s Promus were approved by the FDA in 2008 and 2012, respectively. The characteristics and clinical results of the best candidates of Second-Generation DES are explained below.

#### Endeavor stent

To decrease the issues of the thick strut, inflammation, and late stent thrombosis, Medtronic introduced Endeavor stent, which had a thinner strut of Cobalt-Chromium (mp35 alloy) about 91 μm and was coated with a new antiproliferative drug, Zotarolimus [33] and drug carrier, Phosphorylcholine (PC) as presented in Fig. 2.

The coating thickness is 4.8 μm (1 μm thick PC basecoat, 2–3 μm thick drug layer (90% Zotarolimus, 10% PC)) and 0.1 μm thick PC spray). The quantity of Zotarolimus loaded onto the stent is 1.0 μg/mm^2^ [34]. The drug release profile of Endeavour demonstrated a bit fast release kinetics as 95% of the drug was released within the first 15 days [35].

Endeavor has been widely studied for Clinical trials and exhibited excellent clinical results. The first multi-centric clinical trial was Endeavor I (n = 100) lasted for 12 months. The results of this trial proved its safety and reliability for the treatment of CAD by demonstrating a 2% MACE, 5.4% restenosis rate, and 2% TLR. It was a pilot study and provided support for the larger clinical trials of Endeavour.

The second clinical trial of Endeavour was conducted on a larger population (n = 1197) in 2003. The trial duration was nine months, and its objective was to demonstrate the efficacy and safety of Endeavour DES. The clinical outcomes of the study were compared to the DRIVER (Medtronic) bare metal stent. The results demonstrated that Endeavour is safer and better than BMS by exhibiting (Endeavour vs Driver) MACE = 7.3% vs 14.4%, TVF = 7.9% vs 15.1% and stent thrombosis = 0.5% vs 1.2% [36]. Throughout the study, no excessive edge stenosis, aneurysm formation, or late acquired malapposition were found.

After the successful first-in-man evaluation and comparison with BMS, the Endeavour stent was compared to DES. ENDEAVOR III trial was conducted to compare the safety and efficacy of Endeavour stent with First generation Cypher (Sirolimus eluting stent). It was a prospective, multicentre, single-blind, randomized trial in which 436 patients were enrolled at 29 sites in the US. It was a long-term study that lasted for five years. The trial results demonstrated overall 5.2% patient death in the case of Endeavour and 13.0% in the case of Cypher. The ratio of cardiac death was 0.3:2.8%, stent thrombosis 0.7:0.9 and TVF = 8.1:6.5 [37]. Initially, higher angiographic late lumen loss was observed in Endeavour; however, the rates of clinical restenosis beyond the protocol-specified angiographic follow-up period remained stable with ZES compared with the rates for SES, resulting in similar late-term efficacy.

The endeavor was introduced to solve the issues of first-generation stents and proved its efficacy and safety through various trials. The death rate and MACE were decreased as compared to first generation stents. In conclusion, Endeavour cleared the path for upcoming products and opened gates for new platforms and polymer drug combinations. In 2007, Medtronic introduced another version of the zotarolimus stent called the Endeavor® Resolute. The major difference between the Endeavor Resolute stent and the Endeavor stent is polymer. The Endeavor Resolute stent uses a polymer called BioLinx™ which is the first polymer designed specifically for DES. Besides polymer, the drug and metal substrate was the same as Endeavor.

#### Xience

Xience is one of the best stents ever introduced in the global stent market, encompassing the best possible features a stent can have and consequently demonstrating excellent clinical results in every trial. The metallic platform of Xience is made up of CoCr and has 81 µm strut thickness. The polymeric coating of Xience is double layered:

- The first layer is a base coat which is composed of PBMA
- The second layer is PVDF‐HFP combined with Everolimus (100 µg/cm^2^) [38].

The total thickness of the coating is 7.8 µm, and the drug layer is 5.3 µm thick [39]. The stent design is a nonlinear link and open cell makeing it conformable to the vessel [40], as presented in Fig. 2.

Xience has been widely tested clinically, and proven its safety in each trial. In these clinical trials, Xience demonstrated exciting results of a 0% restenosis rate. To date, Xience DES has been clinically studied in 49 randomized trials containing more than 50,000 patients. SPIRIT I was the first trial of Xience, which was conducted in 2003. In this study, 60 patients were enrolled, and the feasibility and performance of the XIENCE were assessed. The results of 6 months follow up demonstrated that Xience effectively suppressed the neointimal growth at six months compared to its identical bare metal stent. After that, SPIRIT II and III were conducted, and Xience proved its superiority throughout the follow ups. A few clinical trials other than SPIRIT are given below in Table 5.

**Table 5:** Different trials of Xience DES

Trial Name	Stents	Duration	Stent Thrombosis Rate (%)	Reference
ABSIRB II	BVS vs. Xience (n = 501)	4 years	2.8 vs. 0	[41]
ABSORB China	BVS vs Xience (n = 480)	2 years	0.9 vs. 0.0	[42]
STOPDAPT	Xience (n = 1522)	1 year	0	[43]
TWENTE	Endeavor Resolute vs Xience (n = 1391)	1 year	0.58% and 0% (*p* = 0.12)	[44]

Xience is a standard in the stent world because of its superior results and clinical safety. It has shown superior results up to 5 years and remarkably, with a 0% restenosis rate and low MACE. The superiority of Xience over bioresorbable stents is explained in Table 6.

**Table 6:** ABSORB II Trial of Xience vs Bioresorbable stent

End POINTS	1 year	2 year	3 year	4 year	Reference
MACE	4.8% vs. 3.0% (*p* = 0.35)	7.6% vs. 4.3% (*p* = 0.16)	10.5% vs. 5.0% (*p* = 0.04)	11.1% vs. 5.6%	[45]
ST	0%	1.54% vs. 0%	1.8% vs. 0%, *p* = 0.19	2.8% vs. 0%	[45]

Xience has not only been compared with first generation stents but also with second and even third generation stents. In each trial, it demonstrated excellent results. PVDF-HFP inhibited the inflammation, and neointimal hyperplasia, and Everolimus inhibited the cell growth and helped in vessel healing.

#### Promus element

Promus Element (Boston Scientific) revolutionized the cardiovascular industry by introducing a new stent platform called Platinum Chromium (PtCr). It has 81 μm strut thickness coated with the Everolimus drug. The coating of Promus is similar to Xience (PBMA/PVDF-HFP layer of 7 μm thickness), as shown in Fig. 2. The amount of Everolimus loading is 100 mg/mm^2^, and 80% of the drug is released within 30 days, and after 120 days, it becomes non-detectable in the blood stream [46].

Promus Element has proved its safety and effectiveness through five different clinical studies. PLATINUM Workhorse (n = 1530) was a prospective, single arm, multicentre, and observational study which proved the safety and effectiveness of Promus DES by demonstrating 0.9% MACE. PLATINUM Small Vessel and PLATINUM Long Lesion demonstrated ideal results of 0% MACE. PLATINUM QCA and PROMUS Element Plus studies demonstrated 1% and 0.4% MACE, respectively. PROMUS Element Plus was the Post US approval study in which 52 clinical centers and 2681 participants were involved.

Promus and Xience are similar in every aspect except for their metallic platforms. Thus, the clinical trial PLATINUM was conducted to compare both stents. The five years results of this trial are given in Table 7:

**Table 7:** Promus DES vs Xience DES

End Point	Promus (%)	Xience (%)	Reference
Target lesion failure	9.1	9.3	[47]
Cardiac death	2.6	3.5	
Myocardial infarction	3.4	3.2	
Target vessel failure	13.1	12.4	
Stent thrombosis	0.8	0.7	

The results demonstrated that the safety of Promus was equivalent to Xience DES. Despite having non-superior results in this trial, Promus has the advantage of a better metallic platform that is more radio-visible than CoCr. This trait makes Promus an obvious choice for a clinician.

## Third generation stents

The introduction of a first-generation stents (Cypher & Taxus) revolutionized the interventional cardiology field, but second-generation stents (Xience, Promus) are the gold standards of the stent market because they not only solved the problems of first-generation stents (Inflammation, restenosis) but also gave decreased mortality rate. Second generation stents gave very promising results; however, their long-term clinical trials indicated the issue of late thrombosis, very late stent thrombosis, delayed healing, and inflammation at later stages. These issues are associated with permanent polymers of stents which do not erode over time and stays there even all the drug housed by the polymer is eluted [48], becoming the cause of initiation of inflammatory response triggering many other cellular cascades of many other foreign body responses and reactivating restenosis process. Hence, the use of biodegradable polymer is recommended to exclude the issues aroused by the use of the permanent polymer.

The third generation of stents was introduced enclosing stents with either coated with biodegradable polymer or stent without polymer known as polymer free stents.

**Fig. 3:**
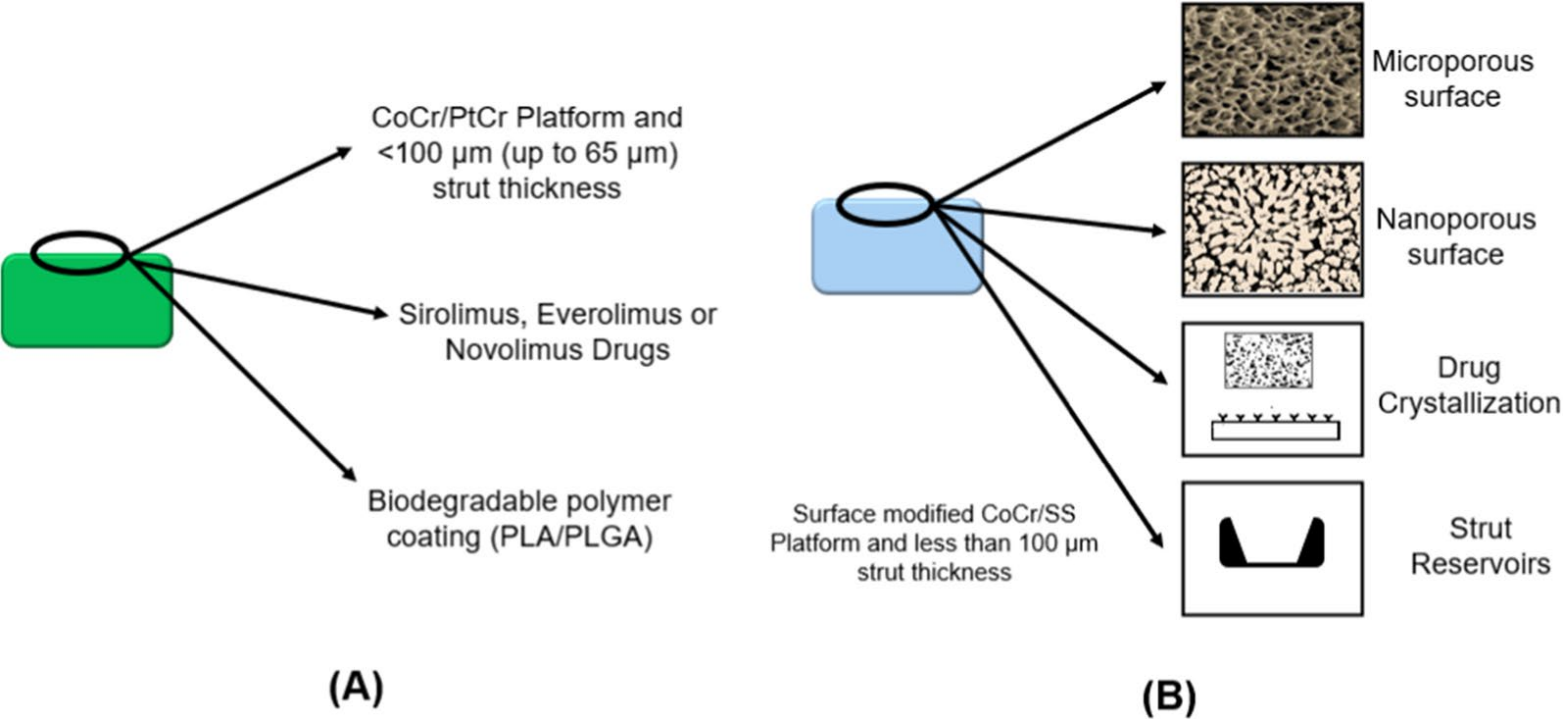
Attributes of 3rd Generation Stents **A** Biodegradable polymer coated DES **B** Polymer free DES

The characteristics of these stents are shown in Fig. 3:

### Metallic platforms

In 3rd Generation DES, metallic platforms of previous generations were used. These stents have stainless steel, CoCr, and PtCr platforms. Stainless steel was reintroduced in this generation as in the previous generation in which only CoCr alloy was used. Among all the three generations of metallic stents, 3rd generation has the most variations among the stents systems because of the use of all three metallic platforms including stainless steel, CoCr, and PtCr. However, PtCr was used only in biodegradable coated stents while the other two platforms are used both in biodegradable coated stents and polymer free stents because they provide greater provision of surface modifications required to hold the drug in case of polymer free drug eluting coatings.

### Drugs

Third generation of DES houses the largest variations of stents because it has gathered all the drugs and platforms in one repository. All members of the Limus family can be found here. Moreover, a new member of the Limus family called BioLimus (also known as Umirolimus) was introduced. Biolimus is a macrocyclic lactone and a highly lipophilic derivative of Sirolimus drug, introduced by Biosensors International and Terumo. It has properties of Sirolimus, i.e., anti-inflammation and anti-proliferation, but its release kinetics are better than Sirolimus. Furthermore, sirolimus was used in both biodegradable and polymer free stent coatings, while Everolimus has only been used in biodegradable polymeric stents.

### Polymers

Third generation stents have a coating of biocompatible and biodegradable polymers. The most common polymers used are polylactic acid (PLA), Poly-d,l-lactic acid (PDLLA), polyglycolide (PLG), and poly(D,L-lactic-co-glycolic acid) (PLGA). These polymers degrade in hydrolytic conditions and because they are biocompatible; their released moieties do not cause much harm.

PLA is a monomer of L-lactide or D-lactide. The degradation rate of PLA is controlled by many factors, i.e., surface area to volume ratio, coating thickness, and porosity. PLA is a biodegradable polymer that converts into carbon, methane, water, and biomass after degradation [13].

**Fig. 4:**
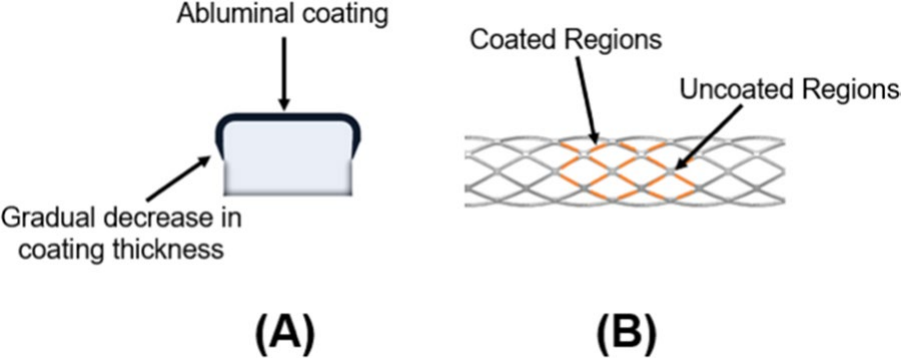
New stent coating technologies **A** Abluminal coating **B** Gradient coating

In addition to the use of various new drugs and polymers in 3rd generation of drug eluting stents, new coating technologies were also introduced. Terumo introduced a gradient style of coating in which the areas which experienced the highest physical stress were devoid of coating to avoid the coating failure at the specific area, which may result in serious inflammatory issues in later stages. Only the middle portion of struts was coated to eliminate the threat of cracking. In this connection, Boston Scientific again marveled the world with their futuristic approaches by introducing the thin coating strategy and coated the stents abluminally with gradient decrease in coating thickness from the middle section towards the edges, as shown in Fig. 4.

### Commercially available Biodegradable Polymer coated Stents (3G-A)

The list of biodegradable polymeric coated drug eluting stents is given in Table 8.

**Table 8:** Commercially available stents coated with biodegradable polymers

Stent	Platform	Drug	Strut thickness (µm)	Polymer	Coating thickness (µm)	Degradation time (months)	Rate of drug release
Biomatrix alpha (Biosensors)	CoCr	Biolimus	88–84	PLLA	10	6–9	60% in 90 days
Ultimaster (Terumo)	CoCr	Sirolimus	80	PDLLA-PCL	15	3–4	90% in 90 days
Yukon Choice PC (Translumina)	316L	Sirolimus	87	PDLLA	5	6–9	More than 80% in 30 days
Synergy (Boston Scientific)	PtCr	Everolimus	74–81	PLGA	4	4	50% in 30 days
Orsiro (Biotronik)	CoCr	Sirolimus	60–80	PLLA	7	14	50% in 30 days

**Fig. 5:**
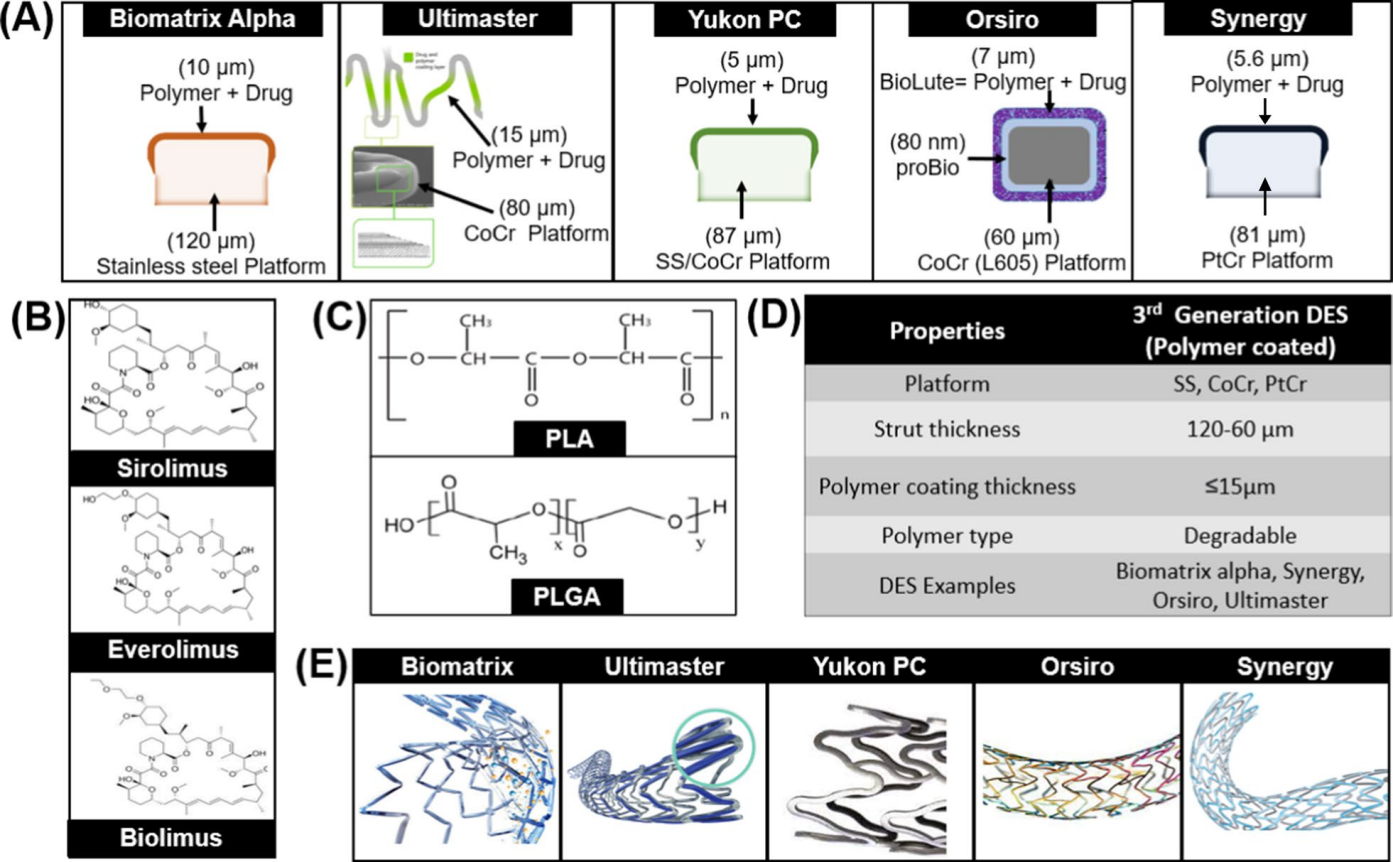
Biodegradable polymer coated DES from 3rd generation **A** Coating of stents **B** Drugs used in 3rd generation DES **C** Polymers used in 3rd generation DES **D** Properties of 3rd generation DES **E** Design of commercially available 3rd generation DES

The properties of stents included in this generation are given below (Fig. 5), and the issues associated with each stent and their clinical outcomes are explained.

#### Biomatrix alpha

Biomatrix Alpha [Biosensors] is CoCr (84 µm) based Biolimus Eluting Stent housed in a 15 um thick PLA matrix. The amount of drug loading is 15.4 µg/mm, as presented in Fig. 5. This stent received CE Market approval in 2015.

Biomatrix has been clinically studied in around ten clinical trials and proved its safety and effectiveness. In the STEALTH trial, its safety and clinical superiority over BMS were demonstrated. Moreover, the LEADERS trial is the first clinical trial of Biomatrix Alpha, where its safety and efficiency were compared with first generation DES. The six months follow-up demonstrated that MACE was 3.8% in Biomatrix. Whereas the 9 months follow up demonstrated 3.9% MACE, 0.8% cardiac death, 1.1% MI, 2.7% TVR. Additionally, the restenosis rate of Biomatrix in 6 months was 2.6% vs. 7.7% of the cypher.

E-biomatrix trials were conducted on varying populations i.e., France, India, Chilli, Canada, and Argentina. The safety and efficiency of Biomatrix for the Asian population was proven in the e-BioMatrix multicentric registry (n = 1189) in India. The 12 months follow up exhibited 0.45% MACE, and only 3 cases of ST were reported [49]. These results proved that Biomatrix stent technology is safe and effective for masses of the various regions and can effectively reduce neointimal proliferation and restenosis [50].

#### Ultimaster

Terumo introduced a unique stent coating technology in Ultimaster DES. It has a unique combination of poly (dl-lactide-co-caprolactone) polymer (PDLLA/PCL) and sirolimus. The polymer is itself a combination of two different polymers such as PDLLA and PCL (9:1). The stent is coated in a unique way that coating makes a gradient in the stent surface as shown in Fig. 5. The polymer is coated only at the struts where less physical stress is applied. The edges of struts experience more stress than the middle portion of struts; therefore, the coating is not applied at the edges. This feature reduces the risk of polymer delamination and cracking [51].

The drug release kinetic of Ultimaster reveals an initial burst release, and after that, there is a slow release with polymer degradation. The polymeric coating degraded after three months and, after then, left with a backbone of BMS.

Ultimaster has been studied in multiple clinical trials. The CENTURY was a six month follow up for Ultimaster which exhibited that the restenosis was 0.9% and late lumen loss was 0.04 mm. In 12 month follow up, 3.8% TLF and 10.9% TVF were observed. Similarly, in two years follow up, Ultimaster showed 5.7% TLF, 2.8% TVF and 0% ST. These results proved that Ultimaster is very safe for CAD with fewer side effects [52].

The CENTURY II was an international study which was conducted in 13 different counties at 58 different medical centres. It was a 12 month follow up trial conducted to compare the results of Ultimaster (a biodegradable polymer) and Xience (a permanent polymer). The comparative results of the trial are given in Table 9.

**Table 9:** Century II trial results (1 year follow-up)

Stent name	Patient number	Cardiac death (%)	ST (%)	TLF (%)	MI-TV (%)	TLR
Xience V	550	1.3	1.3	7.8	2.2	3.9
Ultimaster	551	2.2	0.4	5.3	1.3	2.2

The results of the Century II trial showed no significant difference in results while the outcomes of biodegradable polymers were slightly better than permanent polymer. CENTURY II trial was also conducted on patients suffering from acute coronary syndrome (ACS), and its results are given below in Table 10.

**Table 10:** Century II trial results (ACS sub-study)

Stent name	MI (%)	Cardiac death (%)	ST (%)	TLF (%)	TVF (%)	TLR	Reference
Xience V	4.3	2.3	0	6.5	9.4	3.6	[53]
Ultimaster	2.3	0	0	6.3	6.3	5.5	

There are two other trials, MASTER and e-Ultimaster, in which it has been studied and proved its safety and effectiveness [54]. In conclusion, Ultimaster is safe to use and is an effective treatment option for cardiovascular patients. 

#### Yukon choice PC

Yukon PC [Translumina] is a unique stent with attributes of biodegradable polymeric DES and polymer free DES. It has a microporous surface that is coated with biodegradable Resomer R202S which is Poly (D, L-lactide) with an additional topcoat of shellac resin. The polymer and drug, sirolimus, are coated on the abluminal side of stents and there is no coating on the luminal side of the stent which makes the Yukon more efficient and safer. The microporous surface also shows reduced the rate of restenosis because of lesser contact area with the body tissues.

Clinical trials of Yukon PC demonstrated the reduced risk of early thrombosis and late thrombosis. A four year follow up showed that it reduces the risk of stent thrombosis by 40% and late stent thrombosis by 78%.

Yukon was also studied in ISAR TEST 4 where all three stent generations were compared. In this trial three stents, Cypher, Xience and Yukon PC, were compared. At 30 days, the clinical outcome does not differ much, as TLR for Xience was 4.7% and for Yukon was 4.4%. On the other hand, the rate of stent thrombosis was almost the same. At 12 months, MI for Yukon was 14.4% and for Xience was 13.6%. The results indicated that Yukon had non-inferior results than Xience and superior results than Cypher. The results of 10 year follow up are given in Table 11.

**Table 11:** Comparison of polymer coated DES from all three generations

Stent name	Patient number	MACE (%)	Mortality (%)	ST (%)	TLR (%)	MI (%)	Reference
Cypher	652	54.9	37.2	3.7	22.5	9.1	[55]
Xience V	652	46	30.3	2.5	18.2	7.9	
Yukon PC	1299	47.7	31.8	1.8	20.3	7.7	

Yukon Choice Elite is manufactured in India and considered to be a better version of Yukon Choice PC DES. This stent is made to be suitable for tropical regions and extreme temperature regions. It comes with a temperature sensitive device (Tag Alert) with insulated packaging which is double layered: outer Polystyrene box and inner aluminum packaging. All other properties are the same as Yukon Choice PC. The only difference is of stainless-steel platform instead of cobalt chromium.

#### Synergy

The Synergy [Boston Scientific Corp.] is a stent in which scientists have tried to incorporate all the attributes that an ideal metallic stent should have. A new platform of platinum chromium was introduced through this stent. Synergy is a thin strut (74 µm thickness), Everolimus Eluting Stent. An abluminal coating of biodegradable polymer PLGA (shown in Fig. 5) is used as a drug carrier in this stent system. Approximately 287.2 μg amount of Everolimus is applied on the entire stent with the rate of 1 μg/mm^2^. The stent is coated with 4 µm ultrathin bioabsorbable PLGA. There is no polymer and drug at the luminal side of the stent [56]. The degradation and total absorption time of PLGA is less than four months after the release of the drug [57]. The unique platform of Synergy provides better endothelial cell stent coverage, less inflammation, and reduced platelet adhesion [58]. Synergy got the approval of the FDA in 2020.

Synergy has been studied in multiple clinical studies and demonstrated the lowest relative risk of stent thrombosis. The SWEET registry of Synergy was initiated in 2014 for 1000 patients in Switzerland. The EVOLVE was the first randomized clinical trial in which Synergy was compared to Promus Element. In 2010, when Synergy was at the experimental phase, 291 participants were recruited to investigate the safety of new DES. The results demonstrated that after 30 days, TLF was 0% and 1.1% for Promus Element and Synergy, respectively. At six months, no stent thrombosis was observed in any of the groups [59]. This trial proved that synergy has non-inferior results as compared to Promus Element. EVOLVE trial was a large clinical trial in which 2009 subjects were enrolled at 110 sites in the United States, Europe, Japan, and Brazil. After the successful completion of a first clinical trial, EVOLVE II was started which included diabetic patients as well and results demonstrated non-inferior results for synergy as compared to Promus Element. After three years of follow up, TLF for synergy was 11.1% vs. 10.3% of Promus Element and ST was 0.5% vs. 0.8%, respectively and at five years follow up, TLF was 14.3% and 14.2% and ST was 0.7% vs 0.9%, respectively [60].

These results proved the suitability of PtCr and biodegradable polymer and suggested an excellent combination that can be used as DES coating and should be further explored and improved.

#### Orsiro

The Orsiro [Biotronik, Switzerland] offers a new type of hybrid coating consisting of a passive and an active layer. The passive coating is called PROBIO which is a silicon carbide layer, and its main purpose is to reduce the interaction between stent surface and blood cells. The active coating is called BIOlute which has two components: PLLA polymer and Sirolimus drug. The coating of the Orsiro stent is shown in Fig. 5. Orsiro is a 60 μm thick, ultrathin CoCr (L605) strut covered with a 7.5 μm thick coating layer. It releases 50% drug within 30 days and its polymer degrades in 12–14 months, leaving behind a BMS covered with PROBIO layer [61].

The Orsiro stent was first tested on 30 patients with single de novo lesions. The primary endpoint was 9-month in stent late loss (0.05 ± 0.22 mm). At one year, MACE was 10% with no MI or ST. Following the first-in-man evaluation study, the larger BIOFLOW-II trial compared the Orsiro stent with XIENCE Prime™, a permanent polymer, EES. A total of 452 patients were randomly assigned in 2:1 ratio to be treated with Orsiro (n = 298) or EES (n = 154 patients). Orsiro was non-inferior to EES for the primary endpoint of in-stent late lumen loss at nine months. TLF was similar at one year with no cases of ST in either arm [56].

Orsiro stent demonstrated good results compared to other stents in different clinical trials. BIO-RESORT is a randomized, trial which compared Orsiro with Resolute Integrity and Synergy. Orsiro vs Synergy vs Resolute demonstrated 0.3% vs 0.3 vs 0.3% stent thrombosis and 4%, 4.2% and 4.5% TLF [62]. Another clinical trial BIOSCIENCE compared Orsiro with Xience Prime, and results demonstrated that Orsiro has non-inferior results than Xience. At 12 months follow up, TLF was 6.5% vs. 6.6% (Orsiro vs. Xience). In the subset trial STEMI, TLF was 3.3% vs. 8.7% (Orsiro vs. Xience), indicating superiority over Xience [63].

### Polymer-Free stents (3G-B)

Biodegradable polymer DES was advertised as the future of stents technology. Although it was not superior to second generation stents, it fulfilled its goal of reducing late thrombosis and inflammation. While working on Biodegradable polymers scientists gave birth to a new concept of polymer free stents. Polymer does not have a role in healing or anti-proliferation, so why not eliminate the use of polymer at all.

Polymer free DES was designed to combine the effects of Biodegradable polymer and BMS. The biggest trouble was the adhesion of the drug to the BMS platform and to control the release of the drug. To solve this problem, scientists modified the surface of BMS and converted it into drug reservoirs [64]. Surface modification varied from visible reservoirs to the nano-pores on the surface. These modifications eliminated the need for extra layers of a polymer, leading towards thinner struts. Polymer free DES is a very eye catching and promising technology, but the main challenge for PF-DES has been the attainment of a sufficient level of anti-proliferative drug in the inorganic coating to ensure the inhibition of neointimal hyperplasia and in-stent restenosis [65].

The surface of the stent is modified by various methods. Among these methods, four types are commercially available. The first three strut/surface reservoirs, macropores, and nanopores, can be seen in commercially available stents, but the last one is simply a coating of the drug on the smooth BMS, and this technique is not efficient. Strut/surface reservoirs utilize precise manufacturing processes to accurately inlay the drug into holes or slits in the body of the stent. Some examples include the Janus Flex (Sorin Group), Conor Stent (Conor Medsystems), CoStar (Conor Medsystems), and Nevo (Cordis) [66]. Microporous polymer-free DES contains a modified surface of pits and holes whose size is of the order of microns. The drug is then coated directly on the rough surface, resulting in the micropores being filled and a nominal layer of the drug on top of the surface. The purpose of the micropores is to act as a reservoir for the drug and also to aid adhesion to the stent surface. Nanoporous DES are distinguishable from microporous DES by the nature and size of their pores. They exhibit a bulky porous layer of nano sized pores. This layer of the drug may be obtained electrochemically or through sputter coating techniques. These stents have the advantage of allowing for a higher drug loading capacity. There is another stent in which the pores are arranged in a regular slotted tubular fashion [67]. Table 12 summarizes the characteristics of Polymer free stents.

**Table 12:** Polymer-free Stents and their characteristics

Surface modification technique	Stent	Platform	Drug	Drug quantity ( µg/m^2^)	Release kinetics
Reservoirs					
Laser	NEVO	CoCr	Sirolimus	9.7	80% in 30 days
Sculpted	Janus Flex	SS	Tacrolimus	2.3	50% in 30 days
Drilled	Medtronic’s drug filled stent	CoCr with Tantalum core	Sirolimus	33	–
Pores through microblasting	Yukon	SS\CoCr	Sirolimus	120	60 days
Porous hydroxyapatite coating	VESTAsync	SS	Sirolimus	2.9	30 days

#### NEVO

**Fig. 6:**
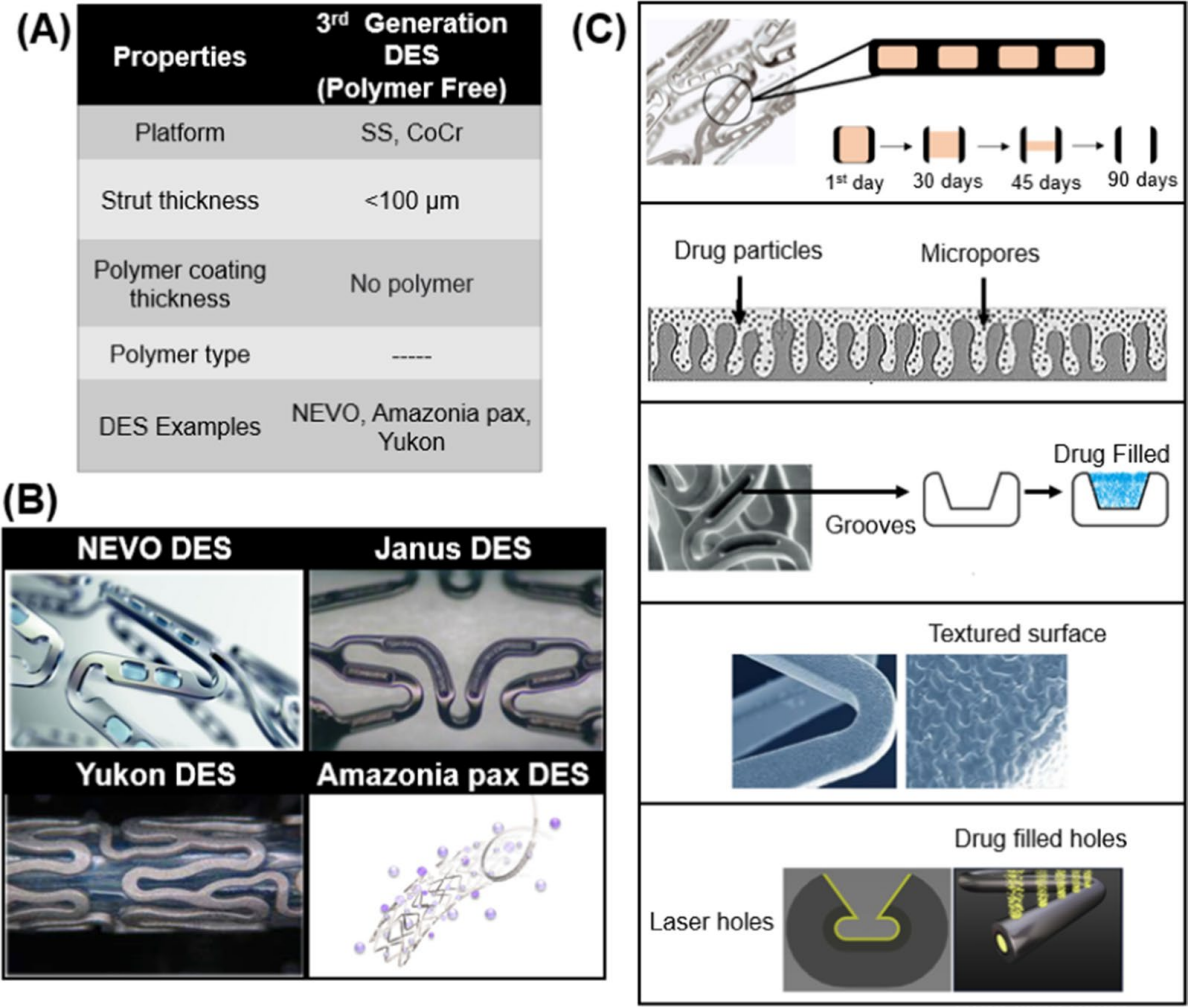
Polymer free third generation DES **A** Attributes of 3rd generation DES **B** Design of commercially available polymer free DES **C** Strategies of drug coating in polymer free DES

NEVO stent [Cordis Corp.] utilized a novel approach of strut reservoirs for the drug delivery which incorporates hundreds of small reservoirs, each acting as a depot into which drug-polymer compositions are loaded. The Cobalt-Chromium platform (99 μm strut thickness) [68] is carved by laser and small reservoirs are created throughout the stent struts as shown in Fig. 6 [69, 70] These struts are filled with Sirolimus along with poly(lactide-*co*-glycolide) (PLGA). The combination of drug and degradable polymer makes it a hybrid instead of a true polymer-free stent. The addition of polymer makes pharmacokinetics more appropriate as they slowly release the drug over the period of four months.

NEVO ResElution-I Trial (n = 394) was conducted to compare the results of NEVO with Taxus Liberte. Results indicated non-inferior results as MI was 2.0% versus 2.6%, TLR was 1.5% versus 3.2% and MACE was 4.0% versus 7.4% [70] and after 1 year follow-up, MACE was 6.1% versus 10.6% and the difference increased after two-years follow-up as MACE was 7.2% versus 13.0% [71]. NEVO stent had a lot of potential for improvement, but it was withdrawn from the market in 2011.

#### Janus

Janus [Sorin Biomedica, Saluggia, Italy] is a polymer-free stent that has a unique feature of surface reservoirs for drug delivery. The stainless-steel platform of stents contains small continuous grooves and holes in it. These groves are present on the abluminal side and suitable for carrying a high amount of drugs. These grooves are filled with an immunosuppressant Tacrolimus drug (Rifamycin analog) which binds to FK-506-binding proteins (FKBPs) just like Sirolimus does [72]. Unlike Sirolimus which makes a complex with FKBP12 and binds to mTOR at 196 amino acids, Tacrolimus makes a complex unit with FKBP12 and further binds to calcineurin which inhibits the activity of serine-threonine phosphatase. This ST-phosphatase activity is important for activating NFATs (nuclear factors of activated T-lymphocytes). These NFATs are responsible for IL-2 and T-cell stimulators [73]. After filling up the reservoirs with the drug, the whole stent is coated with Carbofilm (carbonic layer) which enhances the biocompatibility and reduces the chances of thrombosis. The 50% of the drug is released from the stent within 30 days [74]. The grooves of Janus stent are shown in Fig. 6.

JUPITER II is the clinical trial of Janus stent, which was published in 2005 [75]. The results indicated 7.6% MACE for Janus stent as compared to 10.6% for BMS. Lack of polymer and targeted dosing made the stent more efficient but when it was compared to the results of the RAVEL Trial of Sirolimus-eluting-stents it was not much efficient as their results indicated only 3% MACE [75]. The Janus stent has obtained favorable results in experimental animal studies. In animal studies, it has proven better drug bioavailability and antiproliferative effects but in human trials, it has demonstrated superior results than the first generation but inferior results to second generation stents.

#### Yukon

Transluminal Therapeutics changed the wind of the stent market by introducing the PEARL surface. This is a microporous surface with a density of 1 million pores per cm^2^ and an average micropore depth of approx. 2 µm. They have many products of every attribute, i.e., different metallic platforms, biodegradable coatings and only drug coatings but the common factor is microporous surface. The benefits of this surface have been clinically proven as it showed 25% angiogenic restenosis as compared to the 35% of smooth surface. The surface is roughened by sandblasting technique which is widely used for numerous applications in the world.

Yukon Choice series contains 3 different stents. These stents have stainless steel micro-porous platforms (87 μm strut thickness). Yukon Choice is bare metal stents, and Choice DES and Choice DES^+^ are polymer free stents.

LIPSIA Yukon trial evaluated Yukon stent efficacy and compared it with first generation Taxus Liberte stent. The results were non-inferior results. The Yukon stent was proved to be efficient and effective for the treatment. In this pooled analysis, polymer-free sirolimus-eluting stents were comparable to polymer-based paclitaxel-eluting stents with respect to both angiographic and clinical efficacies.

#### Amazonia Pax

Amazonia Pax [MINVASYS] is a CoCr (L605) polymer free stent (78 µm strut thickness) which is coated with Paclitaxel drug (2.5 µg/mm^2^). This stent does not have any surface modification, i.e., drug reservoirs, grooves or pores, rather has a unique system of drug application on the stent surface which is Microdrop Spray Crystallization. The Paclitaxel drug is Crystallized and applied through a microdrop spray making a 5 µm thick layer. This system gave an optimized drug release profile in which 40% of drug is released on the first day, around 60% by 2 days, 65% by 8 days, and 100% by 45 days. The surface and coating of Amazonia Pax are presented in Fig. 6.

Amazonia Pax has been compared with BMS and Biomatrix in a swine animal model. The results indicated that the Amazonia Pax had a greater lumen area which causes less neointimal hyperplasia response as compared to BMS and Biomatrix response. Results of histomorphometry demonstrated that Amazonia has more neointimal hyperplasia than BMS. The histology results indicated lesser fibrin deposition in Amazonia stent compared to Biomatrix and BMS.

#### VESTAsync

VESTAsync [MIV Therapeutics] is a unique stent with micropores on its surface (Fig. 6). The surface of the stent is coated with a nanometer thin hydroxyapatite coating mixed with Sirolimus drug (55 µg). Hydroxyapatite is a naturally available calcium apatite that makes up the human bones. The in vitro studies have demonstrated that it remains stable for 4 months and completely dissolves after 9 months [76]. VESTAsync has released kinetics similar to CYPHER for the first 30 days; after that the drug release slows down by the rate equal to half of the CYPHER.

VESTASYNC I was the first randomized clinical trial of hydroxyapatite coated DES. The results indicated zero MACE for 2 years and only 1% TLR incident in 3 years. VESTA sync II trial demonstrated 0.39 mm late lumen loss as compared to 0.74 mm of BMS at 8 months [77]. These trials demonstrated the safety of VESTAsync and proved it a major advancement in the field of conventional stent technology.

#### Drug Filled DES

Medtronic has introduced a new DES technology in the coronary stent industry. To make this DES, the inner core material of the stent tube is removed and then filled with the sirolimus (1.1 μg/mm^2^). The small holes are drilled throughout the DES surface (1000 holes per mm^2^) as shown in Fig. 6. The diameter of each hole is 20 μm. To increase the radiopacity, the inner core of DES is made up of tantalum.

Drug Filled DES has completed its pre-clinical and clinical studies. The RevElution trial in 2015 was the first in-man-evaluation for the drug filled DES. For 9 months follow up, the DES demonstrated a 0% restenosis rate and a 2.1% rate of TLF for 100 participants. It has not been commercialized yet.

## Comparison of three generations

The introduction of Cypher caused a sensation in the stent industry because of the benefits of low restenosis and thrombosis rate it provided. Although it is associated with some issues, but it opened a new world in terms of drugs and polymers for scientists. First generation stents with anti-proliferative drugs and durable polymers were able to reduce the rate of restenosis and stent thrombosis, but these gave rise to some other issues, i.e., Late stent thrombosis and very late stent thrombosis. Being pioneers of the technology, these have some shortcomings in terms of stent design, i.e., thick strut, low suitability of the metallic platform, or substandard coatings. These shortcomings were addressed in second generation stents which had better metallic platforms, better drugs, thinner struts, and good polymeric carriers. Second generation stents were the most successful and gave the best results. With the passage of time, a new issue arose in second generation stents that were very late stent thrombosis or inflammation caused by polymeric coatings. These coatings are permanent and do not dissolve or disappear after drug delivery; rather, they stay for a lifetime. Because of vessel pressure, blood flow and moist atmosphere, these coating starts to peel off and cause concerns. These issues are addressed in third generation stents in which biodegradable polymeric coatings are used. This class of stents has shown excellent results which are superior to first generation stents and non-inferior to the second generation of stents. Furthermore, Polymer free class of stents is also superior to first generation stents. There are many trials and experiments that have shown the performance of each generation. ISAR-TEST 4 trial is 10 years follow up trial which compared stents from each generation. The result of this trial is given below in Table 13.

**Table 13:** ISAR-TEST 4 trial

Characteristics	Time interval	First generation (Cypher) (%)	Second generation (Xience V) (%)	Third generation (Yukon PC) (%)
MACE	30 days	–	4.5	4.4
ST		–	0.4	0.4
MACE	12 months	–	14.4	13.8
Cardiac death		–	3.2	2.8
TLR		–	9.4	8.8
ST		–	1.5	1.0
MACE	2 years	–	16	18.8
ST		–	1.4	1.9
TLR		–	9.9	13.5
MACE	10 years	54.9	46	47.7
Cardiac death		37.2	30.3	31.8
MI		9.1	7.9	7.7
ST		2.4	0.8	1.1
TLR		22.5	18.2	20.3

## Fourth generation (bioresorbable stent)

Instead of a metallic platform, scientists developed stents with a polymeric backbone that would disappear once the drug is eluted. This was a unique and creative idea, and many products came into the market, but none of them could stay on market because of their issues. Their major issue was low mechanical strength and drug release profiles. This class is very attractive and has potential. If a scientist could find an ideal candidate in this class, that would be a huge breakthrough in the stent industry.

## Conclusion

Though the introduction of stent technology has revolutionized interventional cardiology and gave improved clinical outcomes; the impediment is still there with stent implantation because of problems aroused in clinical domains. Hence, to overcome these issues, many modifications in every aspect of the stent have been on-going from the time it was introduced in the market till date. The present review debates how drug eluting stent (DES technology) has progressed from thick polymeric drug eluting coating to thin polymeric drug eluting coatings, from durable polymers to biodegradable polymeric coating and finally polymer free coatings. Each generation of stents brought newer and advanced features to solve the problems of the previous generation and, consequently gave rise to new sets of issues, opening new ventures for researchers to explore. Despite all these issues, we are able to achieve reduced stent thrombosis and myocardial infarction in the second generation as compared to first generation drug eluting stents and further reduced the low late stent thrombosis incidents in third generation DES. The second generation of stents gave better results than the first generation in every aspect; still the fear of the existence of a polymer for an eternity lingers on. However, third generation DES solved this issue with the help of biodegradable polymers and even introduced more advanced technology of stents where polymers are no longer needed. Moreover, third generation DES maintained the standard of second-generation DES in every aspect, i.e., reduced risks of stent thrombosis, myocardial infarction, and target lesion failure. We still have the dream of bioabsorbable stents which have the ability to vanish after delivering drugs completely, omitting the issues aroused by polymers. At this moment, however, we have a diverse range of drug-eluting stents that are performing extremely well, and scientists are digging for new avenues for improving DES outcomes.

## Data Availability

Not applicable.

## Abbreviations

BMS: Bare metal stent
CAD: Coronary artery disease
CVD: Cardiovascular disease
DES: Drug eluting stent
FDA: Food and Drug Administration
MACE: Major adverse cardiovascular events
PES: Paclitaxel eluting stents
PLA: Polylactic acid
PDLLA: Poly-d,l-lactic acid
PBMA: Poly n-butyl methacrylate
SIBS: Poly(Styrene-block-IsoButylene-block-Styrene)
PCI: Percutaneous coronary intervention
PEVA: Polyethylene-co-vinyl acetate
PtCr: Platinum chromium
PLG: Polyglycolide
PLGA: Poly (D,L-lactic-co-glycolic acid)
SES: Sirolimus eluting stents
TLR: Target lesion revascularization
TLF: Target lesion failure
TVF: Target vessel failure
